# 

**Published:** 1916-10-07

**Authors:** 


### CONTENTS.

| | PAGE |
|---|---|
| **Comfort versus Appearance** ... ... ... ... ... | 1 |
| **Methods and Machines in Surgery** ... ... | 2 |
| **Hospital and Institutional News** ... ... ... | 3 |
| The Queen at the Royal Free Hospital—The New Hospital for Women, Euston Road—French Decorations for British Women—October 14, 1916—A Red Cross Controversy—A Government Grant for Medicinal Herb Growing?—A Suggested Alteration of Name—Zeppelins as a Source of Income—A Caldow Cot at the London Hospital—The New York Epidemic of Poliomyelitis—An Object-Lesson in Financial Management—A Long Waiting List at Swansea—Death of a Liverpool Eye Surgeon—A Young and Vigorous Institution—Output in Relation to Hours of Work—A Suspicion of Garrotting Dispelled—Women on Asylum Boards—The Medical Society's Plaque from Bolt Court. | |
| **The Delineation of Internal Organs** ... ... | 7 |
| A New Electrical Aid to Diagnosis and Treatment. | |
| **A Red Cross Unit in Serbia...** ... ... ... | 8 |
| The Adventures of the "Berry Mission." | |
| **Life and Death: Some Human Lessons in War-Time** ... ... ... ... ... ... | 9 |
| III.—Childhood and the Children. | |
| **A Visit of Inspection** ... ... ... ... ... ... | 12 |
| **Human Temperaments** ... ... ... ... | 11 |
| XII.—The Philosopher. | |
| **The Matrons' and Sisters' Department** ... | 13 |
| Nurses' Leagues and Matrons' Letters. | |
| Round the Hospitals. | |
| The Reconstituted Committee—Presentation to a Matron—New Matron at Crumpsall—The Value of Linen Guilds—The Sister-Tutor | |
| **The Editor's Letter-Box** ... ... ... ... ... | 15 |
| War Savings Associations—"The British Hospitals Association: A New Departure during War-time"—Mental Nurses in Military Hospitals—The Inflammability of Flannelette: An *Ex Parte* Statement. | |
| **Passing Events and Topics** ... ... ... ... | 16 |
| **British Hospital and Ambulance Work in France** ... .. ... ... ... ... ... | 17 |
| Some Impressions of a Visit. | |
| **Gifts to Hospitals** ... ... ... ... ... | 18 |
| **What's What Column** ... ... ... ... | 18 |
| **Matrons' and Sisters' Appointments** ... ... | 18 |
| **The Institutional Worker** ... ... (Suppl.) | 1 |
| Accuracy in Medicine Giving. | |
| Question for October. | |
| Enquiries and Answers. | |
| The Sick and in Need. | |

